# Longitudinal, EEG-based assessment of sleep in people with epilepsy: An automated sleep staging algorithm non-inferior to human raters

**DOI:** 10.1016/j.cnp.2025.01.001

**Published:** 2025-01-27

**Authors:** Asbjoern W. Helge, Federico G. Arguissain, Lukas Lechner, Gerhard Gritsch, Jonas Duun-Henriksen, Esben Ahrens, Tilmann Kluge, Manfred Hartmann

**Affiliations:** a UNEEG Medical 3450 Allerød, Denmark; b T&W Engineering 3450 Allerød, Denmark; c University of Copenhagen, Copenhagen, Denmark; d AIT Austrian Institute of Technology, Vienna, Austria

**Keywords:** Subcutaneous EEG, ultra long-term EEG, Sleep stage segmentation, Sleep and epilepsy, Sleep parameter estimation, Automatic sleep staging

## Abstract

**Objective:**

There is an unmet need in epilepsy management for tools that measure sleep objectively over long timespans. Subcutaneous EEG is well-suited for the task, but it requires a reliable automatic algorithm. Here, we present and evaluate such an algorithm, and we show clinical examples of how it produces important information.

**Methods:**

A mix of scalp EEG and subcutaneous EEG was used to develop an algorithm to output sleep stages and common sleep parameters. The algorithm was tested on unseen data from 11 healthy subject and 12 people with epilepsy (PwE). Lastly, data (>3months) from three exemplary PwE were analyzed for sleep.

**Results:**

The algorithm proved non-inferior at sleep stage segmentation on data from PwE compared to human raters using scalp EEG. It reached a Cohen’s kappa score of 0.8 [CI 0.78 – 0.83] on healthy subjects and on data from PwE it got to 0.705 [CI 0.663–––0.744] against rater D and 0.686 [CI 0.632–––0.739] against rater E. The three examples showed that useful information can be gained from longitudinal sleep analysis.

**Conclusion:**

Subcutaneous EEG and a sleep algorithm can be employed to effectively review sleep in PwE at a level that is non-inferior compared to human raters.

**Significance:**

This has the potential to make objective sleep parameters available in the clinic as a valuable addition to subjective sleep assessments.

## Highlights

• We developed an algorithm for computing sleep stages and common sleep parameters using subcutaneous EEG.
• Subcutaneous EEG and a sleep staging algorithm proved non-inferior to manual review of scalp EEG for people with epilepsy.
• Examples from clinical data show that longitudinal, automatic sleep staging can provide important clinical information.

## 1 Introduction

Sleep disorders and disturbances are very common in people with epilepsy (PwE), but sleep remains uninvestigated and untreated in most cases (Bergmann et al., 2021, Grigg-Damberger and Foldvary-Schaefer, 2021, Nobili et al., 2021a). Despite numerous scientific findings in recent years on the complex interplay between sleep and epilepsy (Nobili et al., 2022), it has proven difficult for these findings to translate into changes in clinical practice. Once an epilepsy diagnosis has been established, the role of sleep in epilepsy management diminishes or even disappears in most clinical settings (Gibbon et al., 2019). Despite recent expert recommendations to manage sleep disturbances in PwE (Nobili et al., 2021a), clinicians still have limited options to monitor sleep and the impact of clinical decisions over time. This limitation can be partly explained by the inconsistency of patient-reported sleep diaries (Ng and Bianchi, 2014, Robbins et al., 2021), limited time-per-patient (Mahendran et al., 2017), and by the lack of accurate tools to objectively measure sleep for PwE across long periods of everyday life (Danzig et al., 2020).

To address these challenges, there is a pressing need for objective measures of sleep in the context of epilepsy that can complement clinical evaluations and subjective sleep assessments. Utilizing EEG for objective, longitudinal sleep monitoring provides several advantages over other modalities (Imtiaz, 2021). As the cornerstone of the gold-standard in sleep measurements, EEG is a well-established modality within the sleep field, and its use can facilitate the translation of sleep research, which is often EEG-based, into the clinical domain. Scalp EEG-based sleep-staging algorithms often reach superior performance compared to algorithms developed for other modalities (Imtiaz, 2021). However, this comes at the expense of being impractical for real-world long-term monitoring. One proven strategy to make EEG available despite these impracticalities is the use of ultra long-term subcutaneous EEG (sqEEG) monitoring, exemplified by the 24/7 EEG SubQ solution (UNEEG medical, Denmark). This innovative solution allows for continuous collection of EEG data over extended periods, offering new possibilities for investigating sleep in PwE (Duun-Henriksen et al., 2020). To assess sleep from subcutaneous EEG over long periods of time, a robust and reliable automated sleep staging algorithm is required, since manual review would be impractical (Gangstad et al., 2019).

This paper aims to evaluate the performance of a fully automatic sleep staging algorithm for two-channel, ultra long-term EEG against gold-standard polysomnography (PSG). We first describe the algorithm architecture and the dataset used to train and test it. We then demonstrate that the algorithm is non-inferior to human raters, and that it can be used to robustly derive longitudinal sleep parameters. Lastly, we present illustrations to exemplify the potential of assessing sleep in PwE using ultra long-term EEG monitoring, encouraging the scientific community to further investigate the links between sleep and epilepsy.

## 2 Methods

The objective was to develop a fully automatic sleep staging algorithm for two-channel subcutaneous EEG as measured by the UNEEG SubQ (UNEEG medical), which could in turn be used to derive common sleep parameters.

### 2.1 Data

Data from various sources, encompassing both scalp EEG and sqEEG recorded with the 24/7 EEG SubQ solution, were aggregated to form the training, validation and test sets (see Table 1). Acquisition and processing of data from non-anonymized sources were approved by the relevant ethical committees as part of the original studies. All data came from adults and included data from healthy individuals, people with sleep disorders, and PwE. The publicly available scalp data already included sleep stage labels following the standard set forth by the American Academy of Sleep Medicine (AASM) of Wake, N1, N2, N3 and REM (Troester et al., 2023). The private scalp EEG from PwE as well as parts of the private sqEEG from PwE were reviewed specifically for this analysis. All labels relating to sqEEG were annotated on simultaneously recorded scalp EEG, PSG recordings from the healthy subjects (Ahrens et al., 2024), and EMU recordings from the PwE. Annotations were done in accordance with the AASM sleep scoring manual for all PSG recordings, but for recordings from the EMU this was not possible due to missing electrooculograms and sometimes also missing electromyograms. When these channels were unavailable, the scorers tried to follow the manual as closely as possible, which has previously been shown to be an acceptable alternative to scoring PSG recordings (Nguyen-Michel et al., 2021).

**Table 1:** Description of the data sets and the split into training, validation and test sets. * = EMU-based sleep stage labels. PN Cardio: The 2018 PhysioNet/Computing in Cardiology Challenge (Ghassemi et al., 2018, Goldberg et al., 2000); PN Sleep-EDFx: The PhysioNet Sleep-EDF database (Goldberg et al., 2000, Kemp et al., 2000); PN HCM: The PhysioNet Haaglanden Medisch Centrum Sleep Staging Database (Alvarez-Estevez and Rijsman, 2022, Alvarez-Estevez and Rijsman, 2021, Goldberg et al., 2000); PN CAP: The PhysioNet Cyclic Alternating Pattern (CAP) Sleep Database (Goldberg et al., 2000, Terzano et al., n.d.); Clinic Hietzing: non-public 24 h EEG recordings of people with epilepsy; SubQ Healthy 2: (Ahrens et al., 2024).

The dataset was divided into training, validation, and test sets based on the data type, the presence of sleep labels, and whether the data was from PwE (see Table 1). An early version of the developed model was used to generate pseudo-labels for unlabeled sqEEG recordings in the training set. The pseudo-labels were manually curated to identify segments of presumed wake during daytime, that could be a part of the training set.

### 2.2 Algorithm

An algorithm based on a deep learning model was developed to take in two-channel sqEEG and output sleep stages. Heuristics were designed to convert the sleep stages into common sleep parameters such as sleep onset and offset.

#### 2.2.1 Filtering and resampling

EEG data was band-pass filtered between 0.5 and 48 Hz and resampled to 160 Hz, as this sampling frequency combined with specific convolutional layer parameters had proven successful previously.

#### 2.2.2 Spatial and spectral standardization

Linear pre-processing filters were integrated and applied individually across data sets to standardize the spatial and spectral representation (Lechner et al., 2023). These filters enabled both spatial transformations, involving linear combinations of the two input channels, and spectral transformations via finite impulse response (FIR) frequency filtering. Additionally, similar spatial transformations were applied to data to allow for spatial orientation adaptations in the 10–20 system and the various implant positions of the UNEEG SubQ implant.

#### 2.2.3 Modified u-sleep

The sleep scoring model is an adjusted version of the fully convolutional neural network U-Sleep (Perslev et al., 2021). The initial convolutional neural network layers were adapted to integrate the two sqEEG channels in the feature dimension, maximizing the utilization of the available information. The initial kernel size was set to 7 to increase the model's complexity, and the pool and stride sizes for both downsampling and upsampling were adjusted to align with the altered number of input samples per epoch. A dropout layer with a 20 % dropout rate was incorporated at the end of the decoder stack to improve generalization (Srivastava et al., 2014). Positional coding was introduced using three integers to represent the positions of EEG channels within the international 10–20 system. This positional coding was applied after both the encoding and decoding stages.

#### 2.2.4 Correcting short sleep stage phases

A post-processing step was implemented to correct phases of up to ten epochs of continuous REM, N2, and N3 during wake, respectively.

#### 2.2.5 Heuristics to generate sleep parameters

Sleep parameters heuristics were defined to accommodate longitudinal periods, where EEG data is available during all times of the day without indications of lights on and lights out. The same heuristics were used to compute sleep parameters from the algorithm detections and rater annotations. The heuristics were defined as follows:

• **Sleep onset**: A point in time where 2/3 of the next 10 min was sleep, 1/2 of the next 40 min was sleep, and there was a transition from wake epoch to sleep epoch.
• **Sleep offset**: A point in time after a sleep onset where 2/3 of the next 20 min was wake, 1/2 of the next 60 min was wake, and there was a transition from sleep epoch to wake epoch.
• **Nocturnal sleep onset and offset:** The first sleep onset and the last sleep offset of the night.
• **Nocturnal sleep period** (NSP): The period from nocturnal sleep onset to nocturnal sleep offset.
• **Nocturnal sleep period time** (NSPT): The total time of NSP.
• **Nocturnal sleep time** (NST): The time spent asleep during NSP.
• **Wake after sleep onset** (WASO): The time spent awake during NSP.

### 2.3 Performance measure/algorithm evaluation

The sleep stages detected by the sleep staging model were compared with expert annotations, where each 30-second epoch is categorized into True-Positive (TP), True-Negative (TN), False-Positive (FP), and False-Negative (FN). The F1-score (harmonic mean of positive predictive value and sensitivity) and Cohen’s Kappa (*κ*) were calculated for each of the five sleep stages. Additionally, *κ* was calculated for the combination of the four sleep stages and wake (5-Dim). Confidence intervals were calculated by means of bootstrapping.

Sleep parameters calculated from model detections were compared to those calculated from rater annotations.

To demonstrate non-inferiority, we performed a one-sided Wilcoxon signed-rank test evaluating the pair-wise performance difference for both the F1-score and *κ* results. The difference between the mean algorithm vs. rater performance and the mean rater vs. rater performance was computed for each subject and tested against the null hypothesis. We defined non-inferiority margins as 5 %. Non-inferiority is established if 95 % of the subject performance values derived from model detections exceed those derived from rater annotations, adjusted by the specified margin.

### 2.4 Non-physiological detections

The algorithm presented here is implemented in an epilepsy management software (EpiSight Analyzer v2.3, UNEEG medical, Denmark) that prompts a message if the sleep stages distribution is considered outside of physiological limits. Therefore, subjects in the test set for whom the detections exceed these limits will be removed from the rest of the analyses. The physiological limits were defined for REM, N2 and N3 based on previously published observational ranges of PwE (Calvello et al., 2023). They were computed as the upper range value plus half the difference between the upper and lower range values.

### 2.5 Selection of illustrative examples

To illustrate the clinical potential of assessing sleep in PwE using ultra long-term sqEEG monitoring, we selected three examples of PwE in our database that highlight key aspects of the relationship between sleep and epilepsy: (1) seizures occurring almost exclusively during sleep, (2) sleep habit fluctuations over time, and (3) possible effects of antiseizure medication (ASM) on sleep.

Sleep parameters were computed over months for the three PwE. All analyses were done retrospectively (i.e. no clinical decisions were made based upon ultra long-term sleep data). The results are presented in the form of summary tables and charts.

## 3 Results

### 3.1 Discarding non-physiological detections

The computed physiological limits of relative amount of each sleep stage were 34 % for REM, 94 % for N2 and 49 % for N3. Two of the 12 PwE in the test set had a detected relative amount of N3 above the limit and for that reason they were not included in the main analysis.

### 3.2 Sleep staging − performance on healthy subjects

**Fig. 1:**
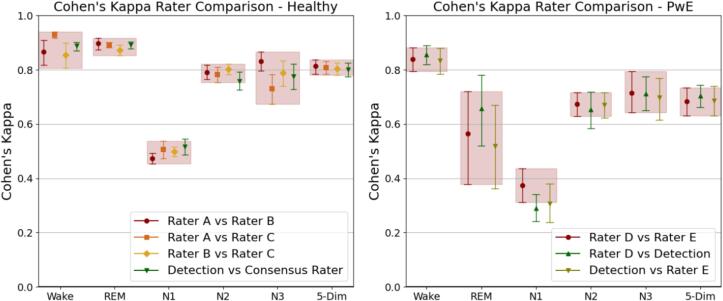
Pair-wise sleep stage label comparison between raters and the algorithm on the data from healthy subjects (left) and people with epilepsy (right). The symbols indicate the mean agreement for the given comparison and the error bares show the confidence intervals. The shaded boxes illustrate the confidence intervals outer limits of the comparison between the raters. 5-Dim is the combined evaluation of the four sleep stages and wake.

The *κ* of the mean pair-wise interrater agreement (IRA) was 0.81 on the healthy data. Non-inferiority for the automatic sleep stage segmentation compared to manual review was confirmed when measuring with F1-score (p-value = 0.016) but was rejected when using *κ* values (p-value = 0.074). The algorithm reached an F1-score of 80.5 % [CI 78.7 % − 82.2 %] and a *κ* score of 0.8 [CI 0.78 – 0.83] when held up against the consensus labels in the healthy dataset. The algorithm performance was withing range of the IRA for all sleep stages as can be observed in Fig. 1. Both the algorithm and the manual reviewers performed best on wake (IRA *κ* = 0.88, algorithm *κ* = 0.89) and REM (IRA *κ* = 0.89, algorithm *κ* = 0.89), and worst on N1 (IRA *κ* = 0.49, algorithm *κ* = 0.52).

### 3.3 Sleep staging − performance on people with epilepsy

The *κ* of the mean IRA between rater D and rater E were 0.68 on the PwE data. The automatic sleep stage segmentation was confirmed to be non-inferior compared to manual review (p-values < 0.01). The algorithm reached an F1-score of 68.7 % [CI 65.1 % − 71.7 %] against rater D and 65.8 % [CI 60.7 % − 71.1 %] against rater E. It reached a *κ* score of 0.705 [CI 0.663–––0.744] against rater D and 0.686 [CI 0.632–––0.739] against rater E. Just as on the healthy data, the algorithm and the manual reviewers performed worst on N1 (IRA *κ* = 0.37, algorithm *κ* = 0.30) and best on wake (IRA *κ* = 0.84, algorithm *κ* = 0.85). There was a mean IRA drop in *κ* value of 0.32 for REM sleep that was reflected in a lower REM sleep performance of the algorithm. REM was also the sleep stage with the most inter-PwE variance in algorithm performance (see Fig. 1, right).

### 3.4 Comparison of estimated sleep parameters

The algorithm estimated NSPT, nocturnal sleep onset and nocturnal sleep offset within 20 min of both raters except for a single outlier, for which the raters also disagreed. This large outlier was also present in the nocturnal sleep onset results, and by closer inspection it could be attributed to the annotation of a small portion of sleep long before the main sleep bout. Both raters and the algorithm had scored sleep at this time (Rater D: 17 m 30 s, the algorithm: 19 m), but one rater had annotated slightly longer sleep (Rater E: 20 m 30 s), thus triggering the nocturnal sleep onset heuristics.

**Fig. 2:**
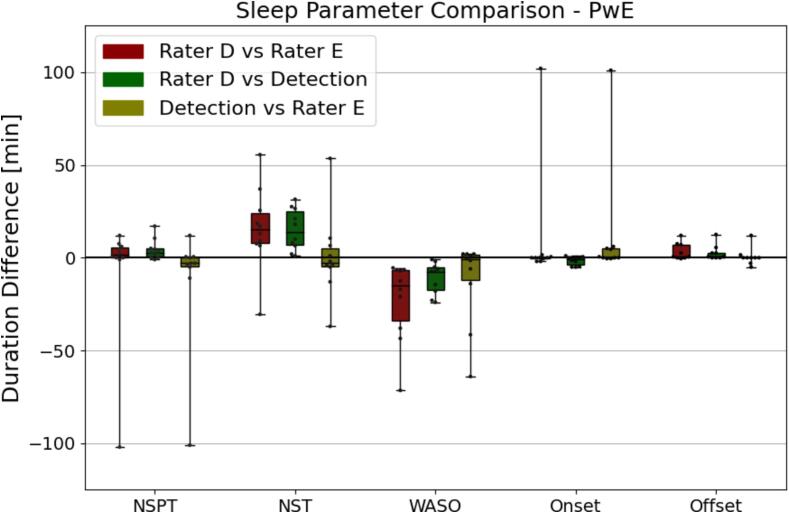
Pair-wise comparison between nocturnal sleep period time, nocturnal sleep time, wake after sleep onset, nocturnal sleep onset and nocturnal sleep offset computed from rater D, rater E, and the algorithm in people with epilepsy (PwE). NSPT, Nocturnal Sleep Period Time; NST, Nocturnal Sleep Time; WASO, Wake after sleep onset.

Visual inspection of Fig. 2 indicates that the algorithm was more in agreement with rater D on WASO and NST than rater D was with rater E. The mean bias and limits of agreement of NST was 15.9 ± 41.49 min for rater D vs. rater E, 15.2 ± 20.71 min for rater D vs. algorithm detections, and 0.7 ± 42.07 min for algorithm detections vs. rater E. The mean bias and limits of agreement of WASO was –22.9 ± 40.43 min for rater D vs. rater E, −10.85 ± 15.69 min for rater D vs. algorithm detections, and −12.05 ± 42.10 min for algorithm detections vs. rater E. Bland-Altman plots for all parameters can be found in the supplementary materials.

### 3.5 Example 1 − seizures occurring predominantly during sleep

**Fig. 3:**
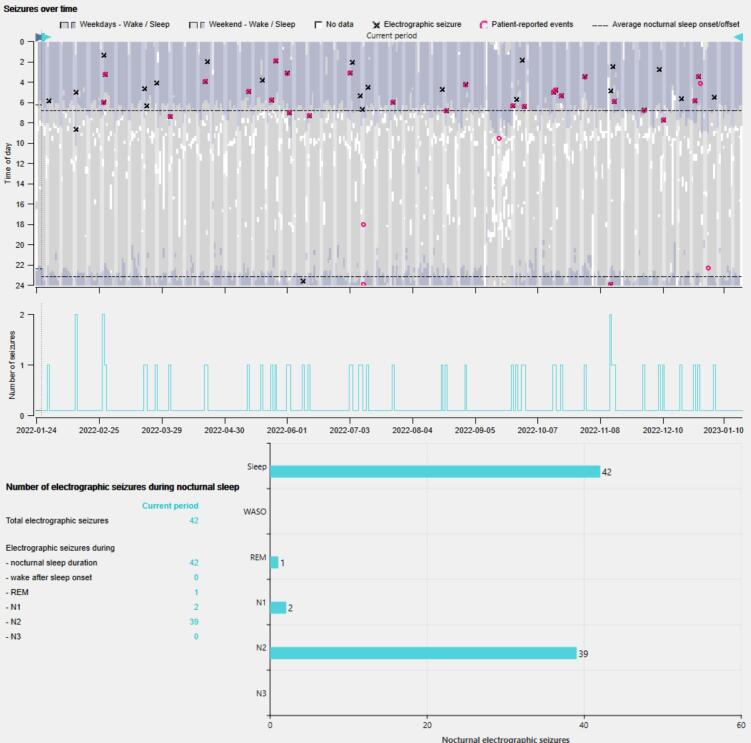
(Top) Electrographic seizures (black crosses), patient-reported events (pink circles) and sleep patterns across 24 h and over time (∼12 months) of a patient who experiences seizures almost exclusively during sleep. Wake and sleep are represented in grey and purple, respectively. Lighter colors represent the weekends. Dashed lines represent the mean sleep onset and offset. (Middle) Daily electrographic seizure count over time. (Bottom) Distribution of which sleep stage nocturnal electrographic seizures occurred in. (For interpretation of the references to colour in this figure legend, the reader is referred to the web version of this article.)

Fig. 3 shows the electrographic seizures, patient-reported events, and sleep patterns across 24 h and over time (∼12 months) of a patient who experiences seizures predominantly during sleep. Most electrographic seizures occurred between 02:00 and 08:00 while the patient was asleep, primarily during N2. Some of the electrographic seizures were accompanied by an immediate self-reported event. The rest of the electrographic seizures are not reported by the patient, likely reflecting that the patient was unaware.

### 3.6 Example 2 − sleep habits fluctuations over time

**Fig. 4:**
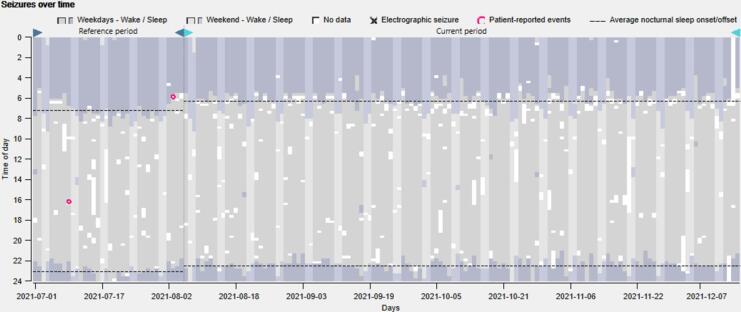
*P*atient-reported events (pink circles) and sleep patterns across 24 h and over time (>5 months) of a patient who has distinct sleep patterns during the summer vacation month in July and different sleep habits during weekends and weekdays. Wake and sleep are represented in grey and purple, respectively. Lighter colors represent the weekends. Dashed lines represent the mean sleep onset and offset. (For interpretation of the references to colour in this figure legend, the reader is referred to the web version of this article.)

Fig. 4 shows the patient-reported events (pink circles) and sleep patterns across 24 h and over time (>5 months) of a patient with epilepsy. The patient did not display electrographic seizures in this period. A considerable change in sleep habits can be observed between the typical summer vacation month (northern hemisphere), July, and the subsequent 4 months, shifting to earlier nocturnal sleep onset and offset. The presence of a weekend effect is also evident, with later nocturnal sleep offset during the weekends (light purple) compared to weekdays (dark purple).

### 3.7 Example 3 − effects of antiseizure medication (ASM) on sleep

**Fig. 5:**
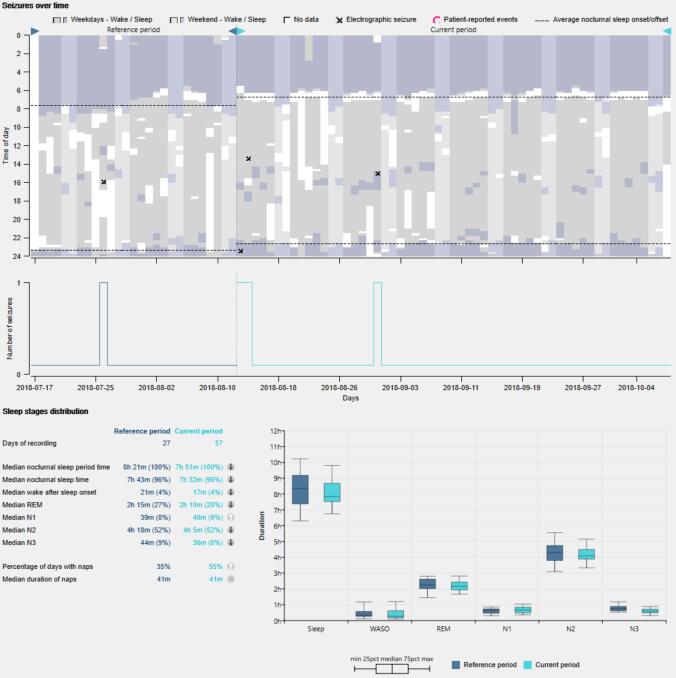
(Top) Electrographic seizures (black crosses) and sleep patterns across 24 h and over time (∼3 months) of a patient who experienced a shift in nocturnal sleep onset and offset from pre to post lamotrigine treatment on the 12 of August 2018. Wake and sleep are represented in grey and purple, respectively. Lighter colors represent the weekends. Dashed lines represent the mean sleep onset and offset. (Middle) Daily electrographic seizure count over time. (Bottom) Nocturnal sleep stage distribution, count of naps and duration of naps. (For interpretation of the references to colour in this figure legend, the reader is referred to the web version of this article.)

Fig. 5 shows the electrographic seizures, patient-reported events and sleep patterns across 24 h and over time (∼3 months) of a person with epilepsy going through ASM titration. After approximately one month, the patient started on lamotrigine, which was up-titrated during the subsequent two months. There was a change in nocturnal sleep time from reference period (before lamotrigine) to the current period (during up-titration of lamotrigine). Two seizures occurred closely after the patient started on lamotrigine.

## 4 Discussion

Sleep is often overlooked in the epilepsy clinic (Gibbon et al., 2019). In this work we have demonstrated that one of the barriers for tackling this issue, namely the lack of objective sleep measures, can be brought down with the use of novel technologies. We showed that an algorithm in combination with sqEEG can provide reliable sleep stages and sleep parameters over ultra long-term periods where manual review is practically impossible. The algorithm proved non-inferior compared to manual sleep scorers for non-discarded PwE, addressing the need for objective sleep measures to accompany subjective sleep assessments.

The agreement between healthy consensus labels and the algorithm detections reached a *κ* value of 0.8, which is on par with algorithms for full scalp EEG data (Gaiduk et al., 2023, Perslev et al., 2021). However, a direct comparison should be made with caution, as the analysis is not made on a benchmark dataset for sqEEG. In fact, the mean pair-wise IRA was high compared to the literature (Nikkonen et al., 2024), which indicates that the healthy test set was easier to score for sleep stages than other data sets. Despite the high performance of the algorithm, non-inferiority could only be confirmed with F1-scores and not with *κ* values in the healthy data set. The algorithm did better than expected on REM sleep given that eye movement can be difficult to see in two-channel sqEEG.

For PwE, the algorithm proved non-inferior to the raters in segmenting sleep stages. The overall performance dropped to *κ* values of 0.705 and 0.686 depending on which rater the algorithm was compared to, and a similar drop could be seen in the pair-wise IRA. This might be reflecting that sleep stage scoring is more difficult in PwE than in healthy because the epileptiform activity obscures the sleep hallmarks (Marzec & Malow, 2003). Moreover, the data from PwE were scored without an electrooculogram, making it more difficult to score REM sleep. This is supported by the large drop in mean IRA of 0.32 on REM sleep from healthy to PwE. Despite the substantial drop in REM sleep performance, N1 sleep was still the hardest sleep stage to classify for the algorithm. However, low N1 sleep performance is not regarded as a problem, because the IRA is also low for N1 sleep, and it also constitutes the smallest part of the sleep stages (Nikkonen et al., 2024). It could thus be argued that the algorithm performed as good as possible by being non-inferior to the raters, since the raters per definition constitute the ground truth. It would be interesting to increase the number of raters in the PwE data to account for the higher scoring variance and to be able to evaluate against a consensus score similar to what has been done for scalp-based algorithms (Guillot et al., 2020).

Just as important as the sleep stages are the sleep parameters, which often represent compressed information of the individual’s sleep habits. NSPT was estimated by the algorithm within 20 min of the rater values, except for a single outlier that indicated a large underestimation. The reason for the outlier was a small sleep segment that both raters and the algorithm had scored as sleep (4-min difference) but only one rater had scored enough to trigger the nocturnal sleep onset. This sleep segment might have reflected an evening nap and not the initiation of nocturnal sleep since it happened long before the main portion of sleep. Unfortunately, the lack of lights off/on labels from the EMU recordings prevented us from discerning between a late nap and the start of nocturnal sleep. Instead, we relied on the time of the day when selecting the time intervals for analysis. This is a technical challenge that is common in real-life monitoring but not in controlled PSG or EMU studies.

Total sleep time —comparable to NST — and WASO are parameters that are often reported by both consumer- and medical-grade wearables and nearables for measuring sleep. Limits of agreement for NST and WASO was lower for the presented algorithm compared with similar values published on healthy subjects using other modalities, indicating better alignment with the raters (Dong et al., 2022, Kainec et al., 2024). More studies are needed to assess how these devices perform on data from PwE.

Two PwE were discarded from the analysis because the relative amount of N3 detections exceeded physiologically meaningful limits in the software that the algorithm was developed for. The reason for the excessive amount of detected N3 might be attributed to frequently reoccurring high amplitude, delta range spike-and-wave trains for these two PwE (see Suppl. Mat. Fig. 8). The limits were implemented because epileptiform activity can significantly disturb physiological EEG sleep patterns in PwE. The limits are a tool to ensure consistency and reliability of the algorithm which are essential for health care personnel to adopt algorithms, especially black box algorithms, into their work routines (Asan et al., 2020). The alternative would be not to use algorithms for ultra long-term data, which is not an option since it is practically impossible to manually review such large datasets for sleep stages in a clinical setting due to limited time (Mahendran et al., 2017). Of course, it would be more optimal if the algorithm functioned equally well for all PwE, but that would require not only a lot more annotated epilepsy data, both public and private, but also more clear descriptions of how to interpret the AASM scoring manual for epileptiform EEG.

### 4.1 Clinical examples

#### 4.1.1 Example 1

In the first example of a clinical application of ultra long-term EEG monitoring, we depicted the case of a person with epilepsy having seizures predominantly during sleep. PwE with nocturnal seizures often report poor sleep quality, particularly those with uncontrolled epilepsy (Planas-Ballvé et al., 2022). Various epilepsy syndromes (sleep-related epilepsies) are strongly associated with sleep (Nobili et al., 2021b), resulting in higher frequency of ictal events and/or potentiated interictal activity while asleep or shortly after waking up. Having too many nocturnal seizures can lead to arousals or awakenings (Peter-Derex et al., 2020) which disrupt sleep and can impact sleep quality of those who have difficulties resuming sleep once awakened (Ohayon et al., 2010). Interictal activity, such as spikes, can also cause arousals, further contributing to sleep disturbances (Peter-Derex et al., 2020). In addition, secondary generalization of seizures is more frequent during sleep than wake in PwE with temporal lobe epilepsy (Garg et al., 2022), which has clinical implications including a higher risk of sudden unexpected death in epilepsy patients (SUDEP) (Lamberts et al., 2012). In this regard, the ability to monitor both seizures occurrence and sleep through ultra long-term EEG monitoring offers a novel opportunity for more informed decisions in epilepsy management. For example, identifying that the patient’s seizures frequently occur during a specific sleep stage could potentially prompt adjustments in medication type, timing, and dosage, which are factors that may improve seizure control (Guilhoto et al., 2011).

#### 4.1.2 Example 2

The second example illustrated a patient whose sleep habits changed from the summer month of July to the period of August through November, and who had a different sleep pattern in weekends compared to weekdays. Irregular sleep habits are known to be a predictor for seizures, and sleep hygiene guidance is a standard tool in epilepsy management care. PwE perceive their sleep as worse than healthy people when self-reporting, and this is a cause for concern and further investigation to confirm sleep alterations (Bergmann et al., 2021). Self-reported sleep diaries and standardized questionnaires are cost-effective and readily available tools that offer valuable insights into the patient's subjective perception of their sleep quality and patterns (Carney et al., 2012, Fabbri et al., 2021). Importantly, most of these questionnaires are not validated for PwE (Nobili et al., 2021b). Moreover, questions have been raised about the validity of some self-reported sleep measures due to discrepancies between reported and objective measures (Ng & Bianchi, 2014). Nonetheless, it has been argued that this discrepancy might offer additional insights not inherent in the subjective patient reports (Andrillon et al., 2020, Nobili et al., 2022). One advantage of objective sleep measures over self-reports is that they are not biased by memory and thus can also be used as basis for probing the patient’s subjective assessment further back than the immediate past (Robbins et al., 2021). Managing sleep in PwE should be an unavoidable part of managing epilepsy [3] and introducing objective ultra long-term sleep measures could help make that true, for the objective as well as the subjective assessment.

#### 4.1.3 Example 3

The third example shows a patient who experienced a reduction in NST from the reference period (before starting on lamotrigine) to the current period (during up-titration). Lamotrigine has been shown to alter sleep by reducing the amount of slow-wave sleep and causing insomnia for some PwE (Foldvary et al., 2001, Liguori et al., 2021). Many other types of ASM are known to affect sleep in different ways, altering both sleep macrostructure (e.g. sleep stages) and microstructure (e.g. spindles) (Lawthom et al., 2023, Liguori et al., 2021, Roebber et al., 2022, Yeh et al., 2021). Importantly, ASM can have different effects (positive or negative) on sleep quality and daytime dysfunction, and the overall effect is more difficult to foresee in PwE with multi-drug treatments (Lawthom et al., 2023). It has been hypothesized that reducing nocturnal sleep disturbance and daytime sleepiness by adjusting ASM can increase the patient’s quality of life (Akdağ et al., 2024, Gutter et al., 2019, Piperidou et al., 2008). Thus, the changes in the objective sleep parameters associated with lamotrigine shown in the third example could have been used to make data-driven treatment adjustments had the PwE manifested clinical complaints.

## 5 Conclusion

Novel tools like ultra long-term EEG monitoring are creating new possibilities to bring objective, longitudinal perspectives into epilepsy management. In this paper, we have demonstrated that a combined setup of sqEEG and a sleep staging algorithm can score sleep in PwE at the same level as manual raters using scalp EEG data. The setup can produce objective sleep measures in a clinical setting and thereby, break down some of the obstacles that limit the use of sleep insights in epilepsy management. Together with subjective assessments, data from objective sleep monitoring, such as the examples given in this paper, has the potential to affect clinical decisions and to support PwE in the understanding of their own condition. Moreover, the presented approach opens new opportunities to investigate the longitudinal relationship between sleep and epilepsy, e.g. by assessing long-term changes in sleep macro structure or the influence of epilepsy cycles on sleep.

## CRediT authorship contribution statement

**Asbjoern W. Helge:** Conceptualization, Methodology, Software, Formal analysis, Data curation, Writing – original draft, Writing – review & editing, Visualization. **Federico G. Arguissain:** Conceptualization, Methodology, Writing – original draft, Writing – review & editing. **Lukas Lechner:** Methodology, Software, Formal analysis, Data curation, Writing – review & editing, Visualization. **Gerhard Gritsch:** Software, Writing – review & editing. **Jonas Duun-Henriksen:** Conceptualization, Methodology, Writing – review & editing. **Esben Ahrens:** Software, Writing – review & editing. **Tilmann Kluge:** Conceptualization, Writing – review & editing. **Manfred Hartmann:** Conceptualization, Writing – review & editing, Methodology, Software.

## Funding

No additional funding was received for the analysis presented here.

## Declaration of Competing Interest

The authors declare the following financial interests/personal relationships which may be considered as potential competing interests: [AWH, FGA, JDH are full-time employees at UNEEG medical A/S. EA has done an industrial PhD with UNEEG medical A/S. LL, MH, GG and TK have served as paid consultants for UNEEG medical A/S.].
