# Supplementary material for: Longitudinal, EEG-based assessment of sleep in people with epilepsy: An automated sleep staging algorithm non-inferior to human raters
Source: Clin Neurophysiol Pract. 2025 Jan 27;10:30–9. doi: 10.1016/j.cnp.2025.01.001 (PMC11833292; doi:10.1016/j.cnp.2025.01.001)
Supplement: Supplementary Data 1 [file mmc1.docx]

# Supplementary materials

## Elaboration data description

The healthy scalp EEG data consisted of data from a collection of publicly available sleep-annotated PSG datasets from healthy individuals and people with sleep disorders. PSG recordings, conducted during nocturnal sleep, included EEG, electrooculography (EOG), electromyography (EMG), electrocardiography, and oxygen saturation data. For our study, we exclusively employed the annotations and the EEG data recorded according to the international 10-20 electrode system. For the scalp EEG data of people with epilepsy (PwE), we utilized non-public 24-hour EEG recordings from 13 PwE recorded in the epilepsy monitoring unit (EMU) in Clinic Hietzing, Austria. The data was annotated for sleep stages by a trained EEG clinician.

Ultra long-term subcutaneous data, recorded using the 24/7 EEG SubQ device from UNEEG medical, was collected from 76 subjects—34 healthy individuals and 42 PwE. Among the healthy subjects, 22 provided 122 nights with sleep stage labels based on simultaneous PSG recordings (Ahrens et al., 2024; Viana et al., 2021). Data from the remaining 12 healthy subjects consisted of 120 segments of unlabeled 24-hour recordings. For the PwE group, 30 subjects provided 300 24-hour recordings of unlabeled data (Weisdorf et al., 2019). Four of the same subjects (Gangstad et al., 2019) and the remaining 12 PwE contributed 23 nights of data with sleep stage labels based on simultaneous full scalp EEG data from the EMU. The last 12 of these were annotated specifically for this study by two sleep experts.


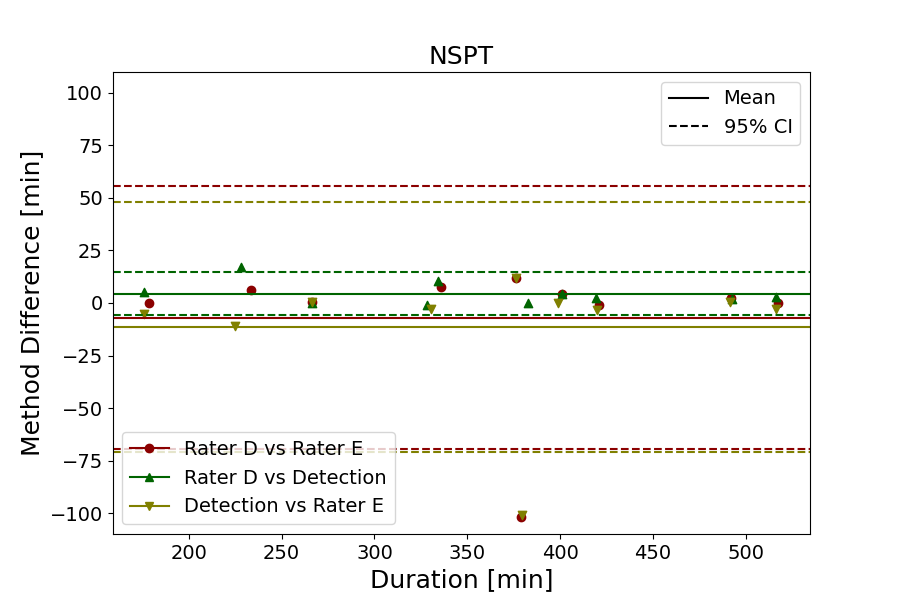

Figure 1. Three-way Bland-Altman plots of nocturnal sleep period time (NSPT) difference between raters and the algorithm in the PwE test set. The solid lines represent the mean bias, and the dashed lines represent the limits of agreement.
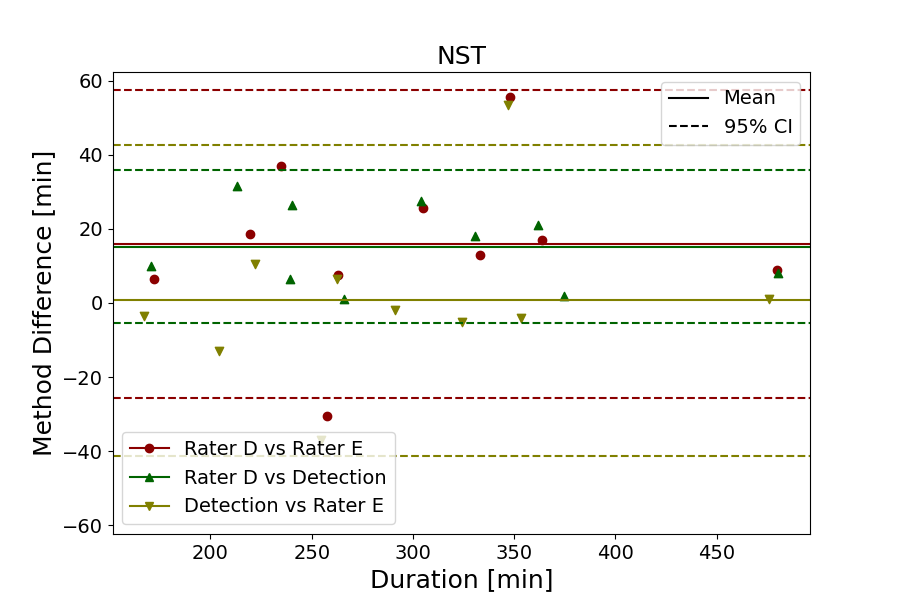

Figure 2. Three-way Bland-Altman plots of nocturnal sleep time (NST) difference between raters and the algorithm in the PwE test set. The solid lines represent the mean bias, and the dashed lines represent the limits of agreement.

.
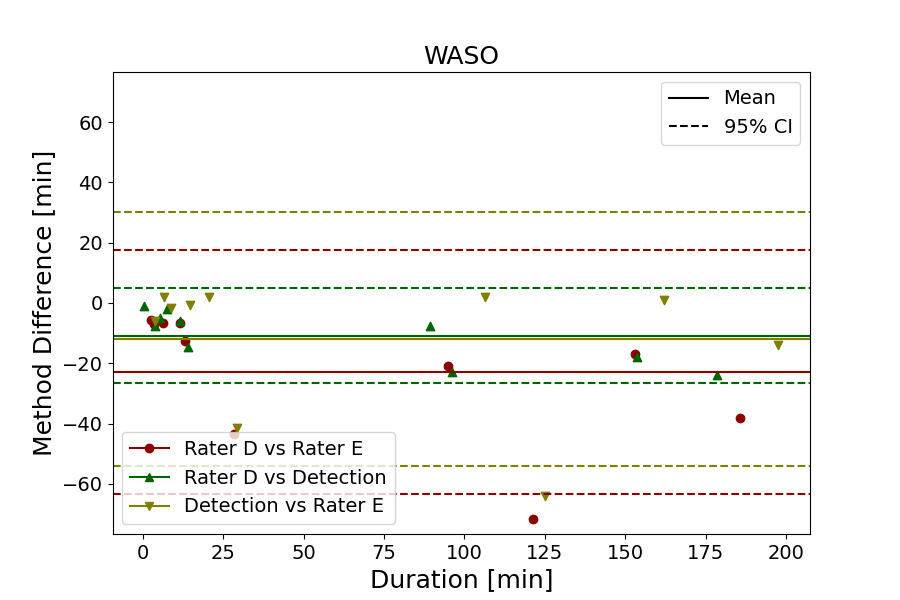

Figure 3. Three-way Bland-Altman plots of WASO duration difference between raters and the algorithm in the PwE test set. The solid lines represent the mean bias, and the dashed lines represent the limits of agreement.

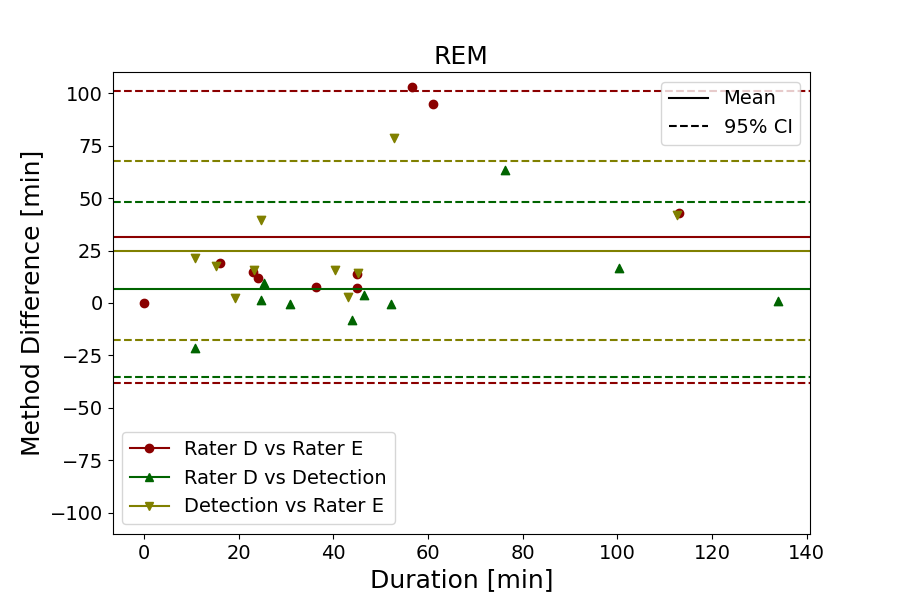

Figure 4. Three-way Bland-Altman plots of REM duration difference between raters and the algorithm in the PwE test set. The solid lines represent the mean bias, and the dashed lines represent the limits of agreement


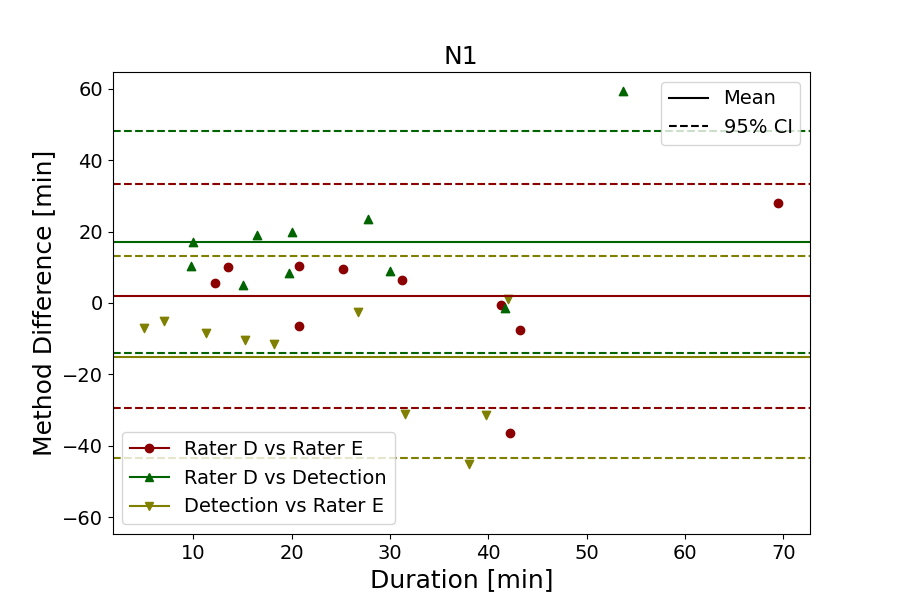

Figure 5. Three-way Bland-Altman plots of N1 duration difference between raters and the algorithm in the PwE test set. The solid lines represent the mean bias, and the dashed lines represent the limits of agreement.

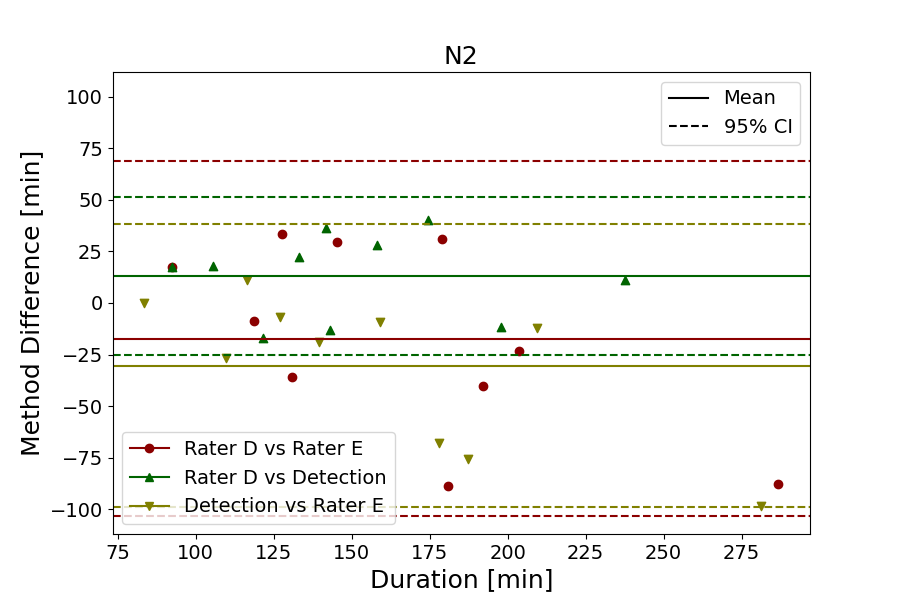

Figure 6. Three-way Bland-Altman plots of N2 duration difference between raters and the algorithm in the PwE test set. The solid lines represent the mean bias, and the dashed lines represent the limits of agreement.
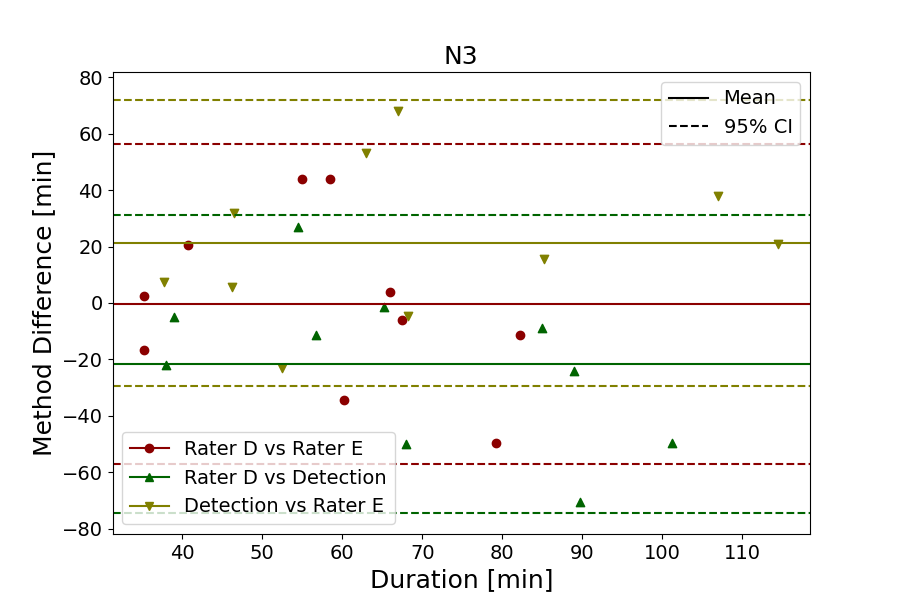

Figure 7. Three-way Bland-Altman plots of N3 duration difference between raters and the algorithm in the PwE test set. The solid lines represent the mean bias, and the dashed lines represent the limits of agreement


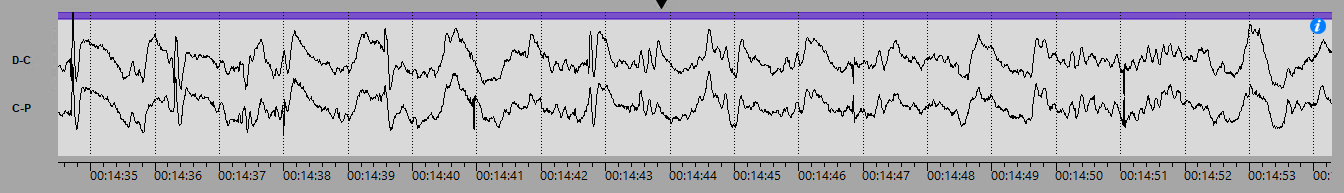

Figure 8. Spike-wave train during N2 for one of the PwE which were discarded because of exceeding the physiological limits of N3 amount.


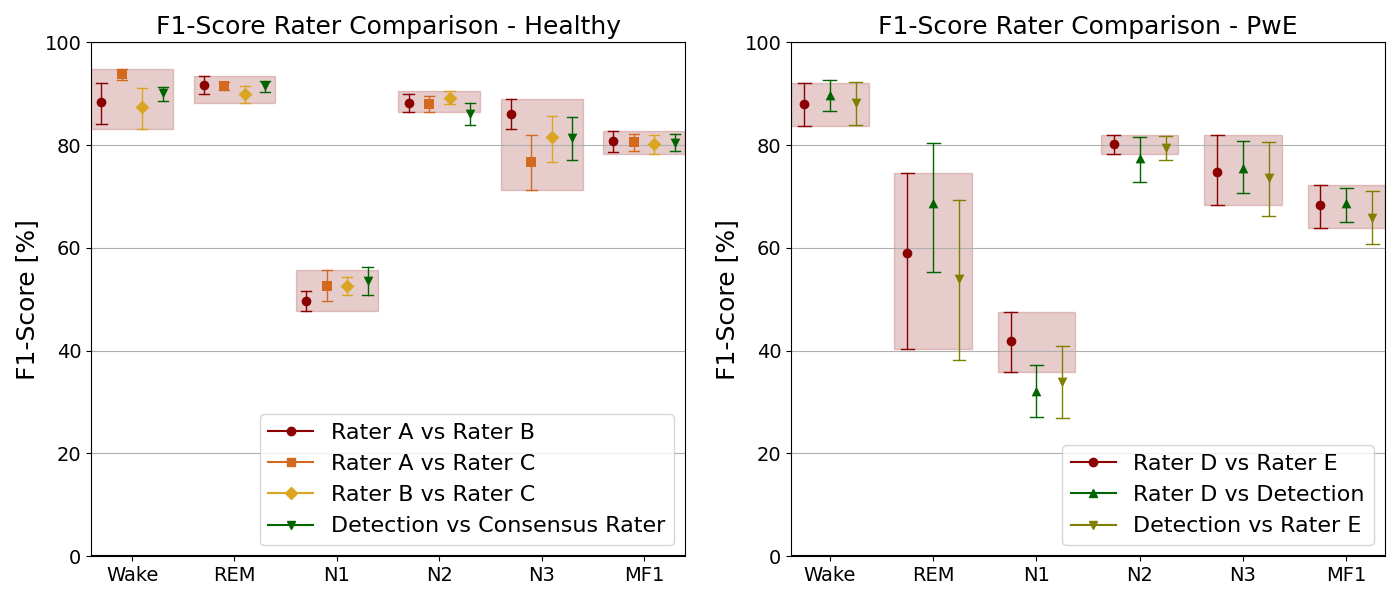

Figure 9. F1-scores from the data set with healthy subjects (left) and people with epilepsy (right). Pair-wise comparison between raters, and between consensus labels and the algorithm detections. The symbols indicate the computed performance for the given comparison and the error bares show the confidence intervals. The shaded boxes illustrate the confidence intervals outer limits of the comparison between the raters.

## Power analysis – estimations using Monte Carlo simulation

Post-hoc analysis of the power in the non-inferiority test were made using Monte Carlo simulations. It was assumed that the underlying distribution of the differences between the algorithm’s performance and the manual reviewer’s performance was a gaussian distribution. It was also assumed that the distribution could be described by the mean and standard deviation of the performance differences reported in the paper. Samples were drawn from the distribution 10000 times and for each time non-inferiority was tested as described in the paper. We found that the three non-inferiority tests which we reported to be significant all had an estimated power above 0.8.
We are aware that the assumptions made in this power analysis can be challenged and for that reason we have chosen to keep these results in the supplementary materials.
